# A steroid‐resistant cockroach allergen model is associated with lung and cecal microbiome changes

**DOI:** 10.14814/phy2.15761

**Published:** 2023-07-04

**Authors:** Nobuhiro Asai, Alexander D. Ethridge, Wendy Fonseca, Kazuma Yagi, Andrew J. Rasky, Susan B. Morris, Nicole R. Falkowski, Yvonne J. Huang, Gary B. Huffnagle, Nicholas W. Lukacs

**Affiliations:** ^1^ Department of Pathology University of Michigan Ann Arbor Michigan USA; ^2^ Immunology Graduate Program University of Michigan Ann Arbor Michigan USA; ^3^ Division of Pulmonary and Critical Medicine, Department of Medicine University of Michigan Ann Arbor Michigan USA; ^4^ Mary H. Weiser Food Allergy Center University of Michigan Ann Arbor Michigan USA; ^5^ Department of Molecular, Cellular and Developmental Biology University of Michigan Ann Arbor Michigan USA

**Keywords:** asthma, cockroach allergen, dysbiosis, gut‐lung axis, microbiome

## Abstract

The pathogenesis of asthma has been partially linked to lung and gut microbiome. We utilized a steroid‐resistant chronic model of cockroach antigen‐induced (CRA) asthma with corticosteroid (fluticasone) treatment to examine lung and gut microbiome during disease. The pathophysiology assessment demonstrated that mucus and airway hyperresponsiveness were increased in the chronic CRA with no alteration in the fluticasone (Flut)‐treated group, demonstrating steroid resistance. Analysis of mRNA from lungs showed no decrease of MUC5AC or Gob5 in the Flut‐treated group. Furthermore, flow‐cytometry in lung tissue showed eosinophils and neutrophils were not significantly reduced in the Flut‐treated group compared to the chronic CRA group. When the microbiome profiles were assessed, data showed that only the Flut‐treated animals were significantly different in the gut microbiome. Finally, a functional analysis of cecal microbiome metabolites using PiCRUSt showed several biosynthetic pathways were significantly enriched in the Flut‐treated group, with tryptophan pathway verified by ELISA with increased kynurenine in homogenized cecum samples. While the implications of these data are unclear, they may suggest a significant impact of steroid treatment on future disease pathogenesis through microbiome and associated metabolite pathway changes.

## 1 INTRODUCTION

Allergic asthma, an inflammatory disease characterized by airway hyperresponsiveness, is one of the most prevalent chronic respiratory diseases, currently affecting more than 300 million people worldwide and is expected to increase to 400 million by 2025. Approximately 250,000 asthma‐related deaths are reported yearly, which are mainly avoidable (Barcik et al., 2020; Christiansen & Zuraw, 2019). The pathophysiological mechanism is still unknown, although several risk factors such as genetic, hygiene, maternal history, and viral infection are associated with the pathogenesis of asthma (Huang & Boushey, 2015). It has been shown that lung and gut microbiome among asthma patients differ from healthy individuals, and dysbiosis may contribute to the pathogenesis and disease progression as well as acute exacerbation (Chen et al., 2020; Durack et al., 2017; Fazlollahi et al., 2018; Goleva et al., 2013; Huang et al., 2015; Li et al., 2017; Pang et al., 2019; Wang et al., 2018; Wang, Chai, et al., 2021; Wang, Lai, et al., 2021; Zhang et al., 2016; Zou et al., 2021). Several asthma phenotypes have been described including atopic, steroid‐resistant, steroid‐sensitive, eosinophilic‐dominant, neutrophilic‐dominant, juvenile‐onset, or adult‐onset. Alteration of the airway microbiome can correlate with asthma phenotype pathogenesis among patients suffering from asthma (Huang & Boushey, 2015; Pang et al., 2019; Zou et al., 2021). Lungs were previously thought to be sterile organs normally absent of microbiome (Bassis et al., 2015; Dickson et al., 2015). However, the lung microbiome has been clearly shown to exist, and it appears that the gut microbiome plays an important role to keep homeostasis with the lung microbiome known as the gut‐lung axis (Asai et al., 2022; Noverr et al., 2004; Yagi et al., 2021).

Cockroach allergen (CRA) is a prevalent antigen influencing an allergic reaction, and 40%–60% of asthma patients in urban and inner‐city areas possess IgE antibodies to CRA (Patel et al., 2016; Sohn & Kim, 2012). Children that show a positive skin‐prick test for CRA allergens are more likely to have difficult‐to‐control asthma and three to four times more likely to have unexpected visits to medical institutes due to asthma‐related events (Gao, 2012). Previous reports indicated that the OVA‐induced asthma murine model showed different microbial compositions compared to naïve mice (Zhang et al., 2021; Zheng et al., 2021; Zheng, Wu, et al., 2022), while modification of the changed microbiome can protect from severe allergic lung disease (Arrieta et al., 2016; Noverr et al., 2004; Raftis et al., 2018; Zhang et al., 2021). The focus of the present studies is whether CRA‐induced asthmatic mice have different microbiomes in the lung and gut compared to normal mice and whether fluticasone (Flut) can impact the microbiome among chronic CRA asthma. These studies indicate that the chronic allergen model that we utilize is resistant to Flut and provides a model for future investigation into treatments with other potential therapeutics for a distinct and difficult to treat patient population. We found that the lung microbiome did not differ due to CRA treatment with or without Flut. However, the gut microbiome was similar in chronic allergic mice compared to normal mice but is changed when animals were treated with Flut. In silico analysis of metabolic processes of the cecal microbiomes demonstrated several significant changes in Flut‐treated animals compared to the nontreated asthmatic group. The tryptophan pathway was one of the most prominent altered and its increased metabolism was verified in the Flut‐treated group by measuring an increased level of kynurenine. Thus, our studies suggest that in a steroid‐resistant model of allergic asthma that there are significant gut microbiome changes that are further altered with steroid treatment protocols that could alter disease processes in future treatment and/or exacerbations.

## 2 MATERIALS AND METHOD

### 2.1 Animals

The Institutional Animal Care & Use Committee (IACUC), University of Michigan, Ann Arbor, approved all animal use protocols, and all experiments proceeded according to IACUC guidelines. Female BALB/c 6–8 weeks of age mice were purchased from Jackson Laboratory. The following standard pathogen‐free conditions were maintained in the Unit for Laboratory Animal Medicine at the University of Michigan: 12‐h light/12‐h dark cycle, 60% humidity, 5L0D PicoLab Rodent Diet, water ad libitum using an in‐line system, sterilization before and after handling, and pine shaving bedding. All caging materials are autoclaved.

### 2.2 CRA asthma model

**FIGURE 1:**
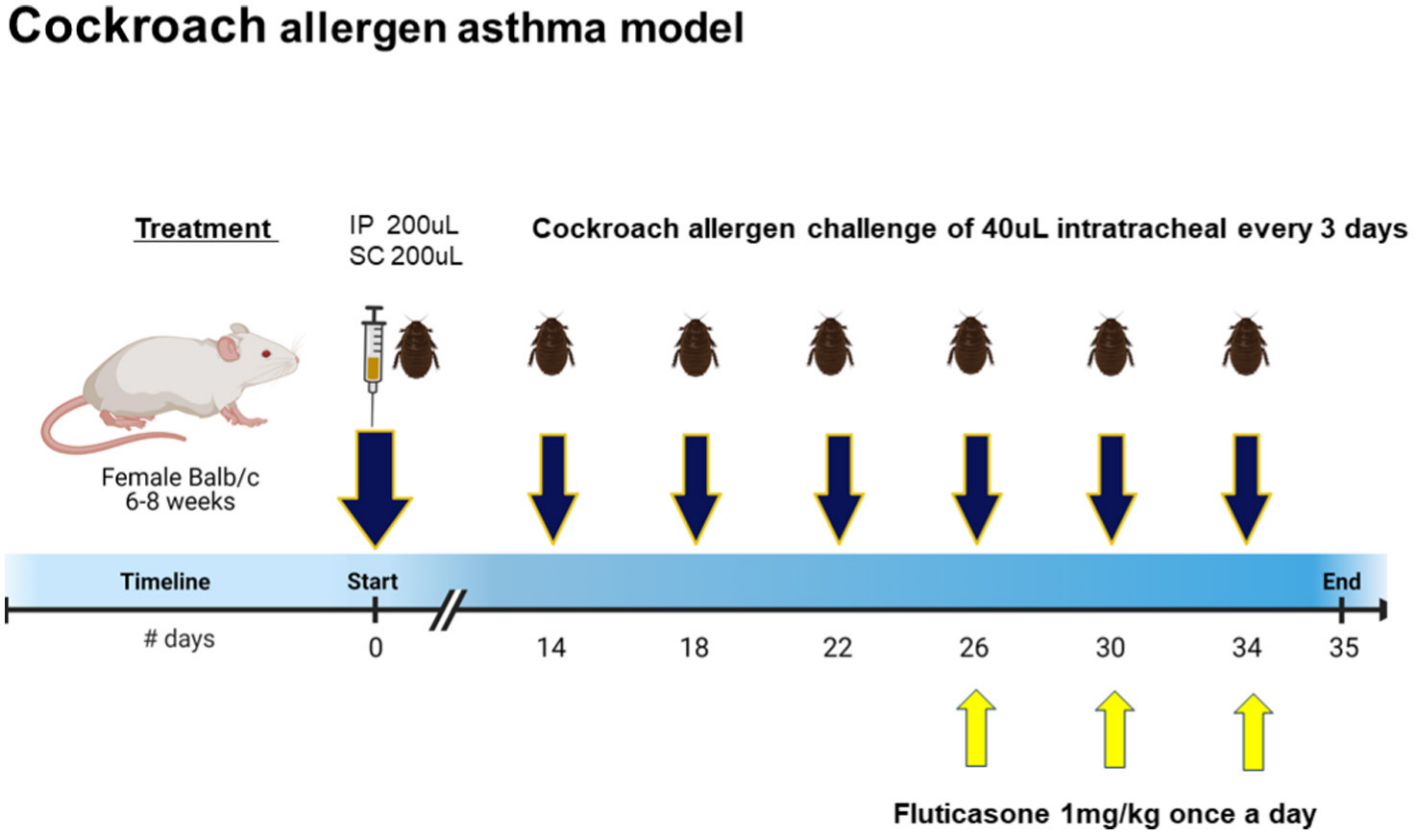
Chronic cockroach allergen model of asthmatic disease. Female BALB/c mice were sensitized to cockroach allergen (CRA) via subcutaneous (SC) and intraperitoneal (IP) injection with a mixture of cockroach allergen and incomplete Freund's adjuvant. Mice were subsequently challenged with CRA via intratracheal instillation to induce allergic inflammation. In some groups, allergic mice were treated therapeutically with fluticasone once/day at the same time as the allergen challenge in a mixture of fluticasone and cockroach allergen.

Mice were sensitized with 500 protein nitrogen units (PNU) of CRA mixed with Incomplete Freund's Adjuvant at a 1:1 ratio via intraperitoneal and subcutaneous injection as previously described (Campbell et al., 1999; Jang et al., 2013; Malinczak et al., 2019). Mice were subsequently challenged intratracheally with 150 PNU of CRA in a volume of 40 μL on Days 14, 18, 22, 26, 30, and 34 after initial CRA sensitization to localize the response to the lung. On Day 35, mice were euthanized, and samples were collected for the indicated analyses after airway hyperreactivity measurement: left lung (flow cytometry or microbiome analysis), right superior lobe (gene expression), right middle/inferior/post‐caval lobes (histology), lung‐draining lymph nodes (CRA restimulation), and cecal tissue (microbiome analysis) (Figure 1). For the analysis of the microbiomes, airway hyperreactivity was not performed prior to sample collected.

### 2.3 Administration of Flut

Flut propionate powder (PHR1702‐200MG, CAS: 80474–14‐2, Sigma‐Aldrich) was dissolved with sterile saline and filtered immediately prior to each administration. Mice were treated intratracheally with 1 mg/kg Flut in a volume of 10 μL on Days 26, 30, and 34 as a mixture with the CRA on indicated days for a total volume of 50 μL.

### 2.4 Lung histology

The middle and inferior lobes of the right lung were perfused with formaldehyde and embedded in paraffin. Five‐micron lung sections were stained with periodic acid‐Schiff (PAS) or hematoxylin/eosin (H/E). Photomicrographs were captured using a Zeiss Axio Imager Z1 and AxioVision 4.8 software (Zeiss).

### 2.5 Airway hyperreactivity

Airway hyperreactivity (AHR) was measured using mouse plethysmography, that is specially designed for the low tidal volumes (Buxco Research Systems), as previously described (Campbell et al., 1999; Lukacs et al., 2001). Briefly, the mouse to be tested is anesthetized with sodium pentobarbital and intubated via cannulation of the trachea with a 18‐gauge metal tube. The intubated mouse was ventilated at a volume of 200 μL at a rate of 120 breaths/min. The airway resistance was measured in the closed plethysmograph by directly assessing tracheal pressure and comparing the level to corresponding box pressure changes. These values were monitored and immediately transformed into resistance measurements using computer‐assisted calculations. Once baseline levels had stabilized and initial readings were taken, a methacholine challenge was given via tail vein (0.350 mg/kg of methacholine) as previously described (Campbell et al., 1999; Lukacs et al., 2001). After methacholine challenge, the response was monitored, and the peak airway resistance was recorded.

### 2.6 Mucus scoring analysis

Slides from PAS‐stained lungs were coded and scored by a blinded observer. Mucus was quantified on a score of 1–4, with 1 = minimal/no mucus; 2 = slight: multiple airways with goblet cell hyperplasia and mucus; 3 = moderate: multiple airways with significant mucus and some plugging; 4 = severe: significant mucus plugging, as previously described (Narayanan et al., 2022).

### 2.7 Flow cytometry

The left lung was removed, and single cells were isolated by enzymatic digestion with 1 mg/mL collagenase A (Roche) and 20 U/mL DNase I (Sigma) in RPMI 1640 containing 10% FCS, and further dispersed via a 18‐gauge needle (10 mL syringe). Red blood cells were lysed, and samples filtered through 100‐micron nylon mesh. Cells were resuspended in PBS and dead cells were discriminated using the LIVE/DEAD Staining Kit (Thermo Fisher Scientific). Cells were washed and resuspended in PBS with 1% FCS prior to Fc receptor blockage using anti‐CD16/32 (Bio Legend). Surface markers were identified using the following monoclonal antibodies (Bio Legend): anti‐Gr‐1 (RB6–8C5), B220 (RA3‐6B2), CD3 (145‐2C11), Ter119 (Ter‐119), CD11b (M1/70), CD25 (PC61), CD45 (30‐F11), CD127 (SB/199), ST2 (DIH9), c‐Kit (2B8), CD90 (53‐2.1), CD4 (RM4‐5), CD3 (17A2), CD8 (53‐5.8), Foxp3 (FJK‐16s), CD69 (H1.2F3) CD11c (N418), MHCII (M5/114.15.2), and CD103 (2E7). The following gating strategies were used for the identification of various immune cell populations: innate lymphoid cell 2 (ILC2) (lineage [CD3, CD11b, B220, Gr‐1, TER119]‐, CD45^+^ CD90^+^ ST2+ c‐Kit+ CD127^+^ GATA3^+^), eosinophils (SSC^high^ CD11b^+^ SiglecF+), neutrophils (SSC^high^ CD11b^+^ SiglecF‐Gr1+), conventional dendritic cell (DC) (CD11b^+^ CD11c^+^ MHCII^+^ CD103^−^), CD103^+^ dendritic cells (CD11b‐ CD11c^+^ MHCII+ CD103^+^), CD4+ T cells (CD3^+^ CD4^+^), CD8+ T cells (CD3^+^ CD8^+^), and Tregs (CD3^+^ CD4^+^ FoxP3+). The Foxp3/Transcription Factor Staining Kit (eBioscience) was used for intracellular staining of Gata3 and Foxp3 to detect ILC2s and Foxp3+ Tregs following staining for surface antigens. Data were collected using a NovoCyte Flow Cytometer (ACEA Bioscience), and analyzed using FlowJo (Tree Star, OR). Using the gating strategies indicated above, the percentage of each cell type within all live single cells was determined. Total cell numbers for each cell type were calculated by applying the percentage to the total number of cells isolated after lung digestion.

#### 2.7.1 Quantitative RT‐PCR

TRIzol reagent was used for lung tissue homogenization and RNA extraction (Invitrogen). Complementary DNA was synthesized using murine leukemia virus reverse transcriptase as indicated by the manufacturer (Applied Biosystem). Real‐time quantitative PCR (qPCR) using Taqman (Thermo Fisher Scientific) primers with a FAM‐conjugated 18S (Mm03928990). Custom primers were designed to measure Muc5ac and Gob5 mRNA levels as described (Miller et al., 2004). Fold change was quantified using the 2‐ΔΔ cycle threshold (CT) method. All reactions were run on a 7500 Real‐time PCR system (Applied Biosystems).

### 2.8 Re‐simulation of lung‐draining lymph node (LDLN) with CRA

LDLN were digested via 1 mg/mL collagenase A (Roche) and 20 U/mL DNase I (Sigma‐Aldrich) in RPMI with 10% FCS for and further dispersed via a 18‐gauge needle (10 mL syringe). Red blood cells were lysed, and samples filtered through 100‐micron nylon mesh. Single‐cell suspensions of lung‐draining lymph nodes (500,000 cells per well) were restimulated with 300 PNU of CRA. The supernatants were collected at 48 h and analyzed for the following cytokines: IL‐4, IL‐5, IL‐13, and IFN‐γ using a Bio‐Plex bead‐based cytokine assay (Bio‐Rad Laboratories).

### 2.9 Enzyme‐linked immunosorbent assays (ELISA)

Kynurenine was quantified from homogenized cecal tissue samples using the Kynurenine ELISA kit (Abnova) according to the manufacturer's instruction.

### 2.10 Microbiome analysis

#### 2.10.1 Sample collection

Left lungs were collected from each mouse and homogenized in 1 mL of sterile water using a Tissue‐Tearor (Biospec Products). The tissue homogenizer was cleaned prior to sample processing using sterile water and ethanol. Control specimens from homogenization post cleaning prior to sample processing and following sample processing were included as procedural controls. Lung homogenates were frozen at −80°C before genomic DNA extraction. Cecal tissue was lightly rinsed to remove cecal contents and snap frozen in liquid nitrogen before bacterial DNA isolation.

#### 2.10.2 Bacterial DNA isolation

Lung homogenates and cecal tissue were homogenized in PowerBead Tubes (Omni International) prior to genomic DNA extraction using the DNeasy Blood and Tissue Kit with a modified protocol that was described previously (Mason et al., 2012). Elution buffer and isolation controls without sample were collected and analyzed for potential sources of contamination.

#### 2.10.3 16s rRNA gene sequencing

The V4 region of the 16 s rRNA gene was amplified from each sample using the dual indexing sequencing strategy developed by Dr. Patrick D. Schloss (Kozich et al., 2013). Sequencing was done on the Illumina MiSeq platform, using a MiSeq Reagent Kit V2 500 cycles (Illumina cat# MS‐102‐2003), according to the manufacturer's instructions with modifications found in the Schloss laboratory standard operating procedure (SOP). Accuprime High Fidelity Taq (Life Technologies) was used instead of Accuprime Pfx supermix. PCR was performed used previously described standard or touchdown conditions for high (cecum) and low (lung) biomass samples, respectively (Dickson et al., 2018; Seekatz et al., 2015). All procedural controls (e.g., elution buffer, isolation controls, homogenization controls, and sterile water) and a positive control mock community (Zymo Research Cat# D6306) were also amplified using PCR as indicated above. All library preparation steps from isolated DNA were performed by the University of Michigan Microbiome Core.

#### 2.10.4 Microbiome analysis

Sequence data were processed and analyzed using the software, mothur (v. 1.42.3) according to the SOP for MiSeq sequence data (Schloss et al., 2009). For each experiment and sequencing run, a shared community file and a phylotype file were generated using operational taxonomic units (OTUs) binned at 97% identity generated using the dist.seqs, cluster, make.shared and classify.otu commands in mothur. Alignment of sequences was performed using align.seqs to the SILVA database (v. 132) according to the mothur SOP. Classification of OTUs was performed using the mothur implementation of the Ribosomal Database Project (RDP) Classifier and the RDP taxonomy training set 16 (Trainset16_022016.rdp) available on the mothur website. Sequences are available via the NCBI Sequence Read Archive data. We performed microbial ecology analysis in R using the vegan (v. 2.5–7) and mvabund (v. 4.2.1) packages. We removed contaminating OTUs identified in procedural controls using the “prevalence” method in the decontam (v.1.12.0) package. For relative abundance and ordination analysis, samples were normalized to the percent of total reads, and we restricted analysis to OTUs that were present at greater than 0.1% of the sample population. All filtered OTUs above this threshold were included in subsequent analysis.

Direct community similarity comparisons within groups were performed using the Bray–Curtis similarity index. Diversity of sequenced communities was analyzed using the Shannon diversity metric. We performed ordinations using Principal Component Analysis on Hellinger‐transformed normalized OTU tables generated using Euclidean distances (Legendre & Gallagher, 2001). We determined pairwise significance of differences in community composition using PERMANOVA with 10,000 permutations using Euclidean distances. *p* values were adjusted for multiple hypothesis testing using the Benjamini–Hochberg method. Family and phylum level abundances were compared via Student's *t*‐test and ANOVA with Holm‐Sidak's multiple comparisons test as appropriate. We performed all analyses in R and GraphPad Prism v 9.3.1. (GraphPad Software Inc., Graphpad Holdings LLC).

#### 2.10.5 Metagenomic inference using PICRUSt2

All OTUs with a relative abundance greater than 0.1% were included in Phylogenetic Investigation of Communities by Reconstruction of Unobserved States (PICRUSt2) analysis (Douglas et al., 2020). A biom file was generated using count data corresponding to all OTUs that met the filtering criteria using the biomformat (v.1.20.0) package. Representative 16s rRNA OTU sequence output from mothur was de‐gapped and pruned to only include filtered OTUs. Prediction of functional abundance was performed as detailed in the PICRUSt2 tutorial (v2.5.0) using default settings. Functional pathway relative abundances were generated after normalization to the total abundance for each sample. Significant functional pathways were identified by LEfSe (Linear Discriminant Analysis with Effect Size) and *p*‐values were corrected for multiple hypothesis testing using the Benjamini and Hochberg correction (Segata et al., 2011).

### 2.11 Statistical analysis

Data were analyzed by Prism v 9.3.1. (GraphPad Software Inc., Graphpad Holdings LLC). Data presented are mean values ± SEM. Comparison of two groups was performed with an unpaired, two‐tailed Student *t*‐test. Comparison of three or more groups was analyzed by ANOVA with a Tukey post‐test. Nonparametric statistical tests were used instead of parametric when appropriate. A *p* < 0.05 was considered significant.

## 3 RESULTS

### 3.1 Steroid resistance in a chronic cockroach asthma model

**FIGURE 2:**
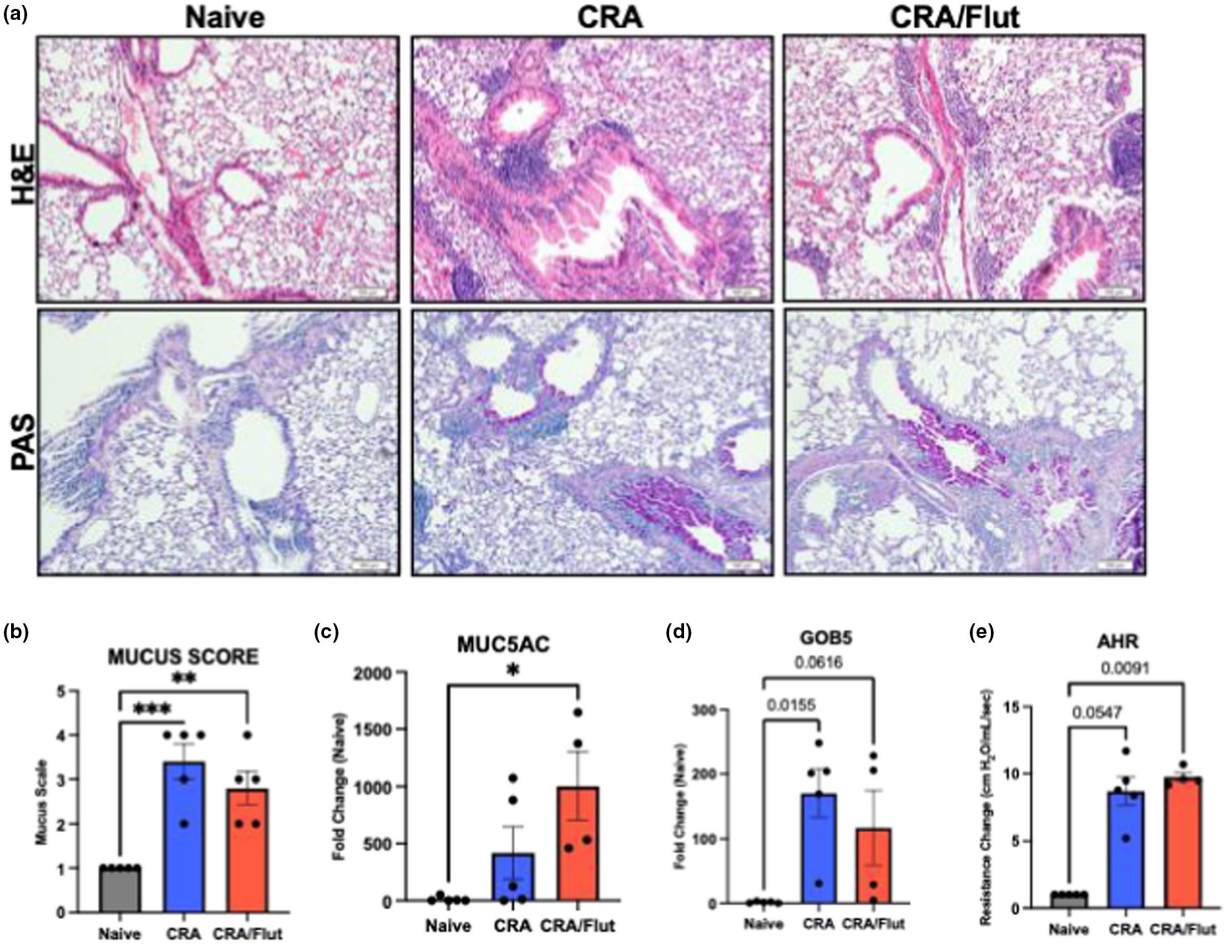
Allergic asthmatic mice are resistant to treatment with fluticasone indicating steroid‐resistance. Naive and allergic animals with or without fluticasone treatment were harvested 24 h after the final allergen challenge. Histopathology was performed on the middle and inferior right lobes by hematoxylin and eosin (H&E) and periodic acid‐Schiff (PAS) staining (a). Assessment of mucus staining was scored by blinded analysis of the PAS‐stained tissue and the expression of the mucin gene muc5ac was measure via qPCR (b, c). Goblet cell hyperplasia was assessed via gob5 goblet cell gene expression (d). The further analysis of airway hyperreactivity (AHR) to a methacholine challenge confirmed the physiologic changes in allergic mice in both the control and fluticasone treated groups (b). Data represent mean ± SEM from five to six mice per group and are representative of two independent experiments with the exclusion of AHR which represents one independent experiment. *p < 0.05, **p < 0.01, ***p < 0.005, ****p < 0.001.

Histological examination of the chronic CRA model of allergic asthma demonstrated tissue and inflammatory changes as indicated by the H/E staining (Figure 2a). Analysis of histopathologic mucus scoring which includes quantification of mucus‐occluded airways in CRA control‐treated and CRA fluticasone‐treated (CRA/Flut) groups showed higher scores than the naive group with no reduction in the Flut‐treated group (Figure 2b). Expression of the mucin gene (muc5ac) was assessed in lungs by PCR to verify mucus scoring and showed that asthmatic Flut‐treated animals had increased mucus production as compared to naïve animals (Figure 2c). Goblet cell hyperplasia which is a typical feature of allergic airway inflammation leading to enhanced mucin production was also assessed via PCR of the gene gob5. Both CRA‐treated groups and without Flut treatment had enhanced expression within the lungs indicating goblet cell hyperplasia (Figure 2d). Measurement of airway hyperreactivity (AHR) was utilized to examine a clinically relevant physiologic parameter of allergic asthma and CRA‐treated animals with and without Flut administration showed a significant increase as compared to naïve animals. However, Flut treatment did not reduce AHR in asthmatic animals (Figure 2e). These initial studies outlined aspects of our chronic modeling that indicate that there are significant pathophysiologic changes which were not attenuated with inhaled Flut treatment such as mucus production, goblet cell hyperplasia and airway hyperreactivity at the assessed timepoint.

### 3.2 Th‐2 cytokines are not altered with Flut treatment

**FIGURE 3:**
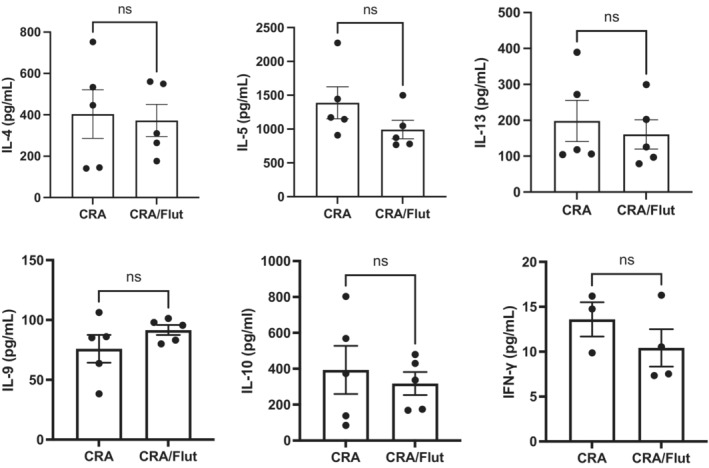
Allergen restimulated lung draining lymph nodes show no alteration of T cell‐derived cytokines in fluticasone treated mice. Lymph nodes were isolated 24 h after the final airway allergen challenge and dispersed into a single cell suspension followed by restimulation with CRA for 48 h. Culture supernatants were isolated and assessed by BioPlex multiplex cytokine analysis to determine the level of cytokine produced due to the allergen challenge. Data represent mean ± SEM from five to six mice per group and are representative of two independent experiments. ns‐not significant.

To evaluate the immune response in the CRA asthma model and to assess whether Flut altered the response, we analyzed the level of allergen‐stimulated cytokines from lung draining lymph nodes of allergic mice (Figure 3). The data indicate that although restimulation of single cell suspensions of lymph node cells led to increases in several Th‐2‐associated cytokines, there were no significant differences in these cytokine levels between CRA and CRA/Flut groups. Naive lung draining lymph nodes were also restimulated and had no detectable levels of cytokine production. These data suggest that along with the lack of changes in the histopathology above, there were no alterations in the T cell associated immune responses due to steroid treatment.

### 3.3 Flow cytometry analysis of lungs from asthmatic mice

**FIGURE 4:**
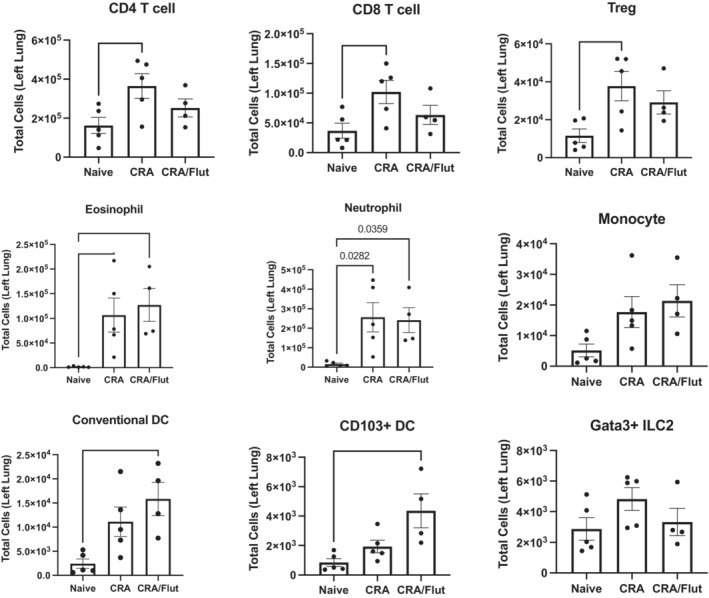
Flow cytometric analyses of lungs of allergen challenged mice show increases in leukocytes and are not altered by fluticasone treatment. The left lung of each mouse was harvested and dispersed by collagenase treatment into a single cell suspension and stained for flow cytometry analysis to identify lymphocyte, granulocyte, and mononuclear cell subsets as designated (see Section 2). Data represent mean ± SEM from four to five mice per group and are representative of one independent experiment. *p < 0.05.

To investigate whether the inflammatory cell accumulation was altered by treatment of Flut the lungs from treated mice were dispersed into single cell suspensions by collagenase treatment. The data indicate that there were increased CD4^+^ T cells, CD8^+^ T cells, Tregs, eosinophils, and neutrophils in the control treated CRA mice as compared to naïve mice (Figure 4). No significant differences in any of the immune cell populations were observed between CRA and CRA Flut‐treated groups (Figure 4). These data suggested that local administration of Flut did not significantly reduce immune cell accumulation overall in allergic asthmatic mice. Interestingly, CD11b + DC and CD103+ DC were elevated in allergic animals treated with Flut compared to the naive mice (Figure 4). Thus, while there were some altered cell populations within the lungs of allergic mice treated with Flut, no significant change was observed in Flut‐treated mice compared to CRA control‐treated mice. These data are consistent with observations in the pathophysiology measurements above furthering supporting that the chronic CRA model utilized is steroid‐resistant.

### 3.4 Pulmonary and gut microbiome changes

**FIGURE 5:**
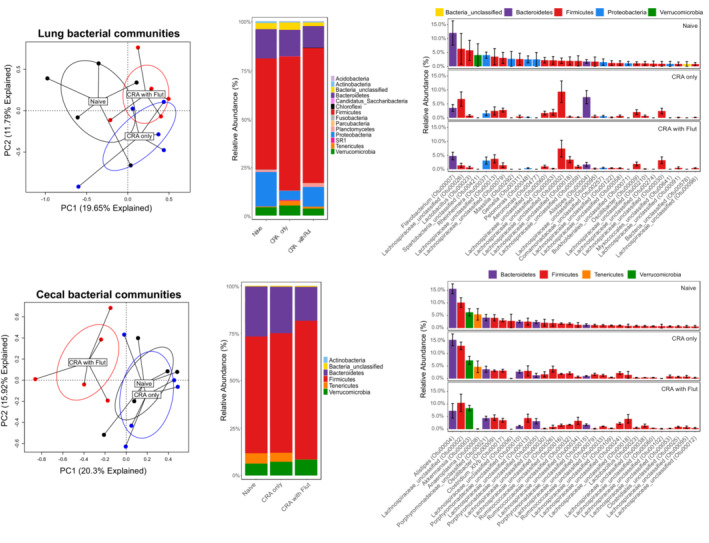
The gastrointestinal microbiome is altered by fluticasone in the steroid‐resistant CRA allergy model while the lung microbiome was unaltered. The left lungs and cecal tissue from naïve or allergic mice were harvested at 24 h after the final allergen challenge and processed for 16s rRNA sequencing. The bacterial communities were compared via principal component analysis and PERMANOVA analyses to determine if there were differences in the communities (a, d). The stacked abundance plots of bacterial phyla (b, e) were further assessed by relative abundance plots based upon OTU assignment at the genus level (c, f) to further investigate differences in the bacterial communities. Data represents five mice per group and are representative of one independent experiment.

An area that has become a center of investigation is the role that mucosal microbiomes have in dictating the severity of disease. To explore whether there were changes in the lung microbiome in a steroid‐resistant asthma model, the bacterial communities were profiled using 16S rRNA amplicon sequencing at the end of CRA model of allergic asthma. The overall composition of the bacterial communities in the lungs were not significantly different from one another (Figure 5a). However, there is clearly a shift in the relative abundance of various phyla and taxa that is induced by CRA and/or CRA/Flut treatment **(**Figure 5b,c **).** The alpha diversity or species diversity within the lung microbiomes did not differ between groups (Figure S1). However, the naive group had higher Bray–Curtis dissimilarity as compared to CRA or CRA/Flut treatment indicating a greater diversity within the group (Figure S1). When examining the microbial composition at the phylum level, the relative abundance of the phylum Verrucomicrobia was increased in the CRA group compared to naive group (Figure S2). With these changes in the composition of the lung microbiome at the phylum level, PICRUSt2 analysis was performed to assess inferred functional pathway abundances using the MetaCyc database and compared using LEFse to assess functional differences. There were no significantly altered pathways as expected by the observation that the overall community compositions were similar in the lungs of control and asthmatic mice regardless of Flut treatment (data not shown).

We next examined the cecal microbiome. The composition of the CRA/Flut group was significantly different from the naive and CRA groups whereas the composition of naïve and CRA groups were very similar **(**Figure 5d **)**. While there was a shift of relative abundance of various phyla and taxa in the CRA and CRA/Flut groups compared to naive mice **(**Figure 5e,f **)**, the overall bacterial diversities did not differ between groups (Figure S3A,B). However, examination of taxonomic changes indicated that the phylum Tenericutes (Figure S4), specifically the family Anaeroplasma in CRA/Flut group was significantly reduced (Figure S5).

**FIGURE 6:**
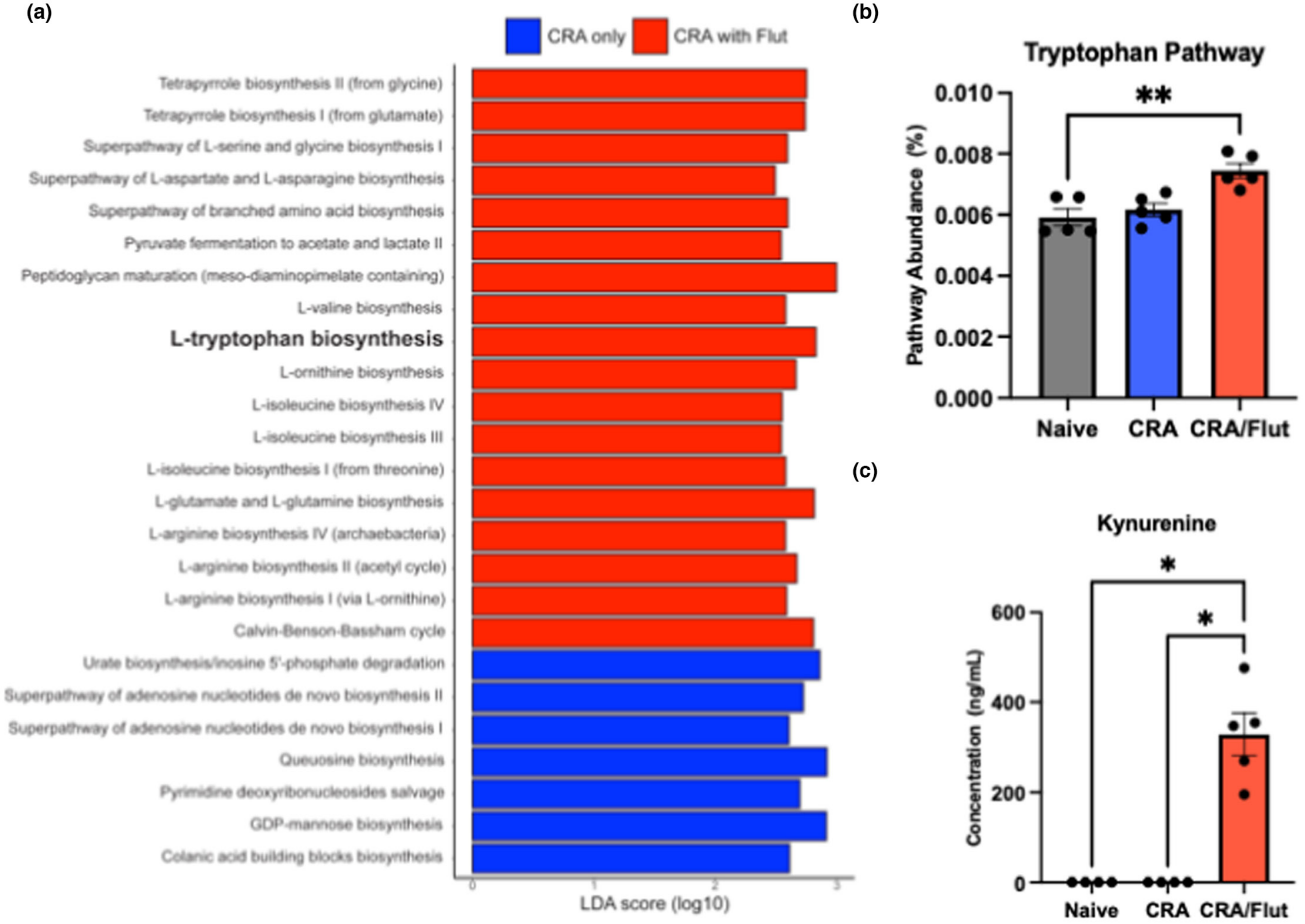
An altered gastrointestinal microbiome induced by fluticasone treatment produces more metabolites of tryptophan biosynthesis. Functional pathway relative abundances were generated after normalization to the total abundance for each sample using Picrust2 metagenomic inference. Significant functional pathways were identified by LEfSe (Linear Discriminant Analysis with Effect Size) and p‐values were corrected for multiple hypothesis testing using the Benjamini and Hochberg correction (a). The tryptophan pathway relative abundance was calculated for each group (b). The tryptophan pathway metabolite, kynurenine, was measured in homogenized cecum samples from mice 24 h after the final allergen challenge by specific ELISA (c). Data represent the mean ± SEM from four to five mice per group are representative of one independent experiment. ****p < 0.001.

To determine whether the differences in the gastrointestinal microbiome led to functional differences, PICRUSt2 analysis was performed. The naive and CRA‐treated control animals had no differences in their inferred metabolomes using principal component analysis which is consistent with their similarity in bacterial community composition (data not shown). However, the CRA control‐treated and Flut‐treated inferred metabolomes differed from one another via principal component analysis (data not shown). Subsequent analyses focused on control CRA and Flut‐treated asthmatic cecal inferred metabolomes for this reason. Interestingly, treatment of the CRA model with Flut was associated with a prediction of increased amino acid synthesis pathways, whereas the control‐treated CRA mice showed enrichment for nucleotide metabolism pathways, colonic acid biosynthesis and GDP‐mannose biosynthesis (Figure 6a, LDA score (log10) > 2). One pathway, tryptophan biosynthesis, was significantly enriched in CRA mice treated with Flut (Figure 6b, one‐way ANOVA, *p* < 0.05). To validate these findings, we analyzed and found an increase in kynurenine, one of the principal breakdown products of tryptophan via ELISA in cecal homogenates (Figure 6c, one‐way ANOVA, *p* < 0.05). Thus, treatment of chronic steroid‐resistant asthma with Flut into the airway led to alterations in the gut microbiome that appeared to further alter the functional metabolic pathways that could influence disease outcomes.

## 4 DISCUSSION

Corticosteroids are one of the most prescribed treatments for those suffering from asthma (Pruteanu et al., 2014). More recently, inhaled corticosteroids have been shown to alter the lung/airway microbiome and may complicate the progression of responses that contribute to future disease severity (Begley et al., 2022; Huang et al., 2022; Martin et al., 2020; Yip et al., 2022). Thus, understanding the consequence and overall impact of inhaled steroids on the local and systemic microbiome during disease may provide important insight into the future studies and how disease may be modified. Using an established model of chronic allergic asthma, the results indicate a steroid‐resistant phenotype in all the immune and pathophysiologic parameters examined. While the control CRA treated animals showed no differences in the lung and gut microbiome as compared to naive animals, chronic asthmatic mice treated with Flut into the airway showed significant changes to the gut microbiome. A previous study using an adjuvant OVA‐induced allergic asthma mouse model found microbiome changes in the gut (Zhang et al., 2021); however, no alternation in the lung microbiome was confirmed. In human studies, changes in the lung microbiome have been observed in patients with asthmat and chronic obstructive pulmonary disease (COPD) treated with steroids; however, the consequence of these changes remain unclear for future responses (Contoli et al., 2017; Hartmann et al., 2021; Huang et al., 2022). Interestingly, the significant difference in the microbial composition of the cecum between CRA and CRA/Flut groups has not been previously described but provides a potential link via the lung‐gut axis. While inhaled corticosteroid (ICS) is considered to be the first‐line therapy for asthma (Pruteanu et al., 2014), some asthma patients are resistant to ICS treatment due to different asthma phenotypes (Guida et al., 2022; Han et al., 2010; Hizawa, 2022). We hypothesize that ICS can alter gut microbiomes in asthma patients who are resistant to ICS therapy and subsequently alter their immune responses, potentially contributing to poor outcomes in difficult‐to‐treat patients suffering from asthma.

While the overall outcome of immune cytokine phenotype did not change, flow cytometry of lung tissue showed an elevation of conventional dendritic cells (DC) and CD103^+^ DCs in asthmatic Flut‐treated animals that is associated with alteration of the gut microbiomes. It has been shown that invasive microorganisms penetrate epithelium to activate DCs and the presence of increased numbers of DCs can lead to enhanced immune activation upon subsequent exposures. Activated DCs express co‐stimulatory ligands to induce CD4 T cells to differentiate into pathogenic effector Th cells (Fujiwara, 2021; Harusato et al., 2015). Recent data have suggested that patients with increased frequency of circulating myeloid, but not plasmacytoid, DC in patients with steroid‐resistant asthmas as compared to those with steroid‐sensitive asthma (Chambers et al., 2018). Thus, the predisposition of steroid resistance may be maintained by increased numbers of “*inflammatory*” DC leading to a maintained or exacerbated allergic environment.

Recent data in asthmatic and COPD patients have suggested that microbiome changes observed in steroid‐resistant patients include organisms that specifically metabolize corticosteroid and may contribute to the resistant phenotype (Durack et al., 2017; Goleva et al., 2013; Huang et al., 2015). In the lung, while there were overall changes the data was not significant and since only the left lobe was used to assess other coincident changes in the lung, use of the whole lung may have provided additional changes due to differential microbiome alterations in different lung regions. It is likely that organisms altered in the lung are not only influenced by the immune environment but also the O_2_ levels in the aerobic environment that is modulated by the increased mucus and changes in pH, two primary drivers of opportunistic environments that influence the microbiome community and can be differentially altered in different regions (Vacca et al., 2020). Interestingly, we found the relative abundance of Anaeroplasmataceae was significantly decreased in the cecum microbiome of the Flut‐treated animals. These genera are considered to play roles in homeostasis by producing short chain fatty acids (SCFAs) that promote barrier function and tolerogenic immune environment (Abdalkareem Jasim et al., 2022; Lyu et al., 2022; Ma et al., 2022; Nguyen et al., 2019; Tchitchek et al., 2022). While these descriptive results indicate changes in microbiome are associated with disease, the influence on the severity is speculative, but likely centers around other contributing factors such as the metabolite profile from the microbiome community (Blachier & Andriamihaja, 2022; Zhao et al., 2022).

An initial attempt to examine how the microbiome changes alter the metabolite profile with PiCRUSt analysis, showed no significant effect in the low biomass environment of the lung, but significant changes in specific pathways in the gut microbiome of Flut‐treated animals. Interestingly, several the amino acid biosynthetic pathways were upregulated and is consistent with recent metabolite observations in asthma patients (Daley‐Yates et al., 2022; Durack et al., 2017). One pathway in particular, tyrosine metabolism, is often altered during inflammatory disease (Vacca et al., 2020; Zhao et al., 2022; Zheng, Parra‐Izquierdo, et al., 2022). In agreement with the PiCRUSt in silico analysis, Kynurenine, a degradation product of tyrosine metabolism, was significantly upregulated in cecal samples of Flut‐treated animals. It has been shown that kynurenine–tryptophan pathway metabolites are associated with several diseases such as neurological disease, mood disorder, cancer, atopic disease, and asthma (Fujiwara et al., 2022; Gostner et al., 2016; Passarelli et al., 2022). Furthermore, severe hypertryptophanemia can disturb immune and neurological homeostasis (Klaessens et al., 2022) and might be a signature of unbalanced immune regulation of allergic diseases. Tryptophan concentrations were also found to be higher in patients with allergic rhinitis (Ciprandi et al., 2010). Licari et al. concluded that IDO pathway metabolites and neopterin production are increased in children with allergic asthma and could be a potential marker of a dysregulated immune response (Licari et al., 2019). One possible explanation is that changes in the gut microbiome by lung administration of Flut impacted kynurenine–tryptophan pathway metabolite regulation and contributed to “steroid‐resistance”.

While an obvious criticism of these studies is centered on its descriptive nature, we highlight that the gut microbiome in chronic steroid‐resistant asthmatic mice are quite different from those in naive mice. Surprisingly, local administration of Flut impacts the gut microbiome as compared to untreated asthmatic mice and highlights enhanced amino acid metabolism pathways verified by measurement of kynurenine in cecal microbiome that represents the tryptophan metabolism pathway. These results provide interesting data that could highlight how the modulation of the microbiome by steroid treatment may potentiate future responses in resistant asthma phenotypes.

## ETHICS STATEMENT

All experiments involving the use of animals were approved by the University of Michigan Institutional Animal Care and Use Committee (IACUC) under protocol #PRO00011224 “Immune mechanisms of Lung disease”. The health of the animals was monitored daily by University Laboratory Animal Research Specialists.

## FUNDING INFORMATION

HHS | NIH | National Heart, Lung, and Blood Institute (NHLBI): Nicholas W. Lukacs, R35HL150682; HHS | NIH | National Institute of Allergy and Infectious Diseases (NIAID): Gary B. Huffnagle, Nicholas W. Lukacs, R01AI138348; HHS | NIH | National Institute of Allergy and Infectious Diseases (NIAID): Nicholas W. Lukacs, P01AI089473.

## Supporting information


Figure S1:

## References

[phy215761-bib-0001] Abdalkareem Jasim, S. , Jade Catalan Opulencia, M. , Alexis Ramirez‐Coronel, A. , Kamal Abdelbasset, W. , Hasan Abed, M. , Markov, A. , Raheem Lateef Al‐Awsi, G. , Azamatovich Shamsiev, J. , Thaeer Hammid, A. , Nader Shalaby, M. , Karampoor, S. , & Mirzaei, R. (2022). The emerging role of microbiota‐derived short‐chain fatty acids in immunometabolism. International Immunopharmacology, 110, 108983. 35750016 10.1016/j.intimp.2022.108983

[phy215761-bib-0002] Arrieta, M. C. , Sadarangani, M. , Brown, E. M. , Russell, S. L. , Nimmo, M. , Dean, J. , Turvey, S. E. , Chan, E. S. , & Finlay, B. B. (2016). A humanized microbiota mouse model of ovalbumin‐induced lung inflammation. Gut Microbes, 7, 342–352. 27115049 10.1080/19490976.2016.1182293 PMC4988436

[phy215761-bib-0003] Asai, N. , Kato, H. , & Mikamo, H. (2022). The pathophysiological mechanisms of COVID‐19 and host immunity, with emphasis on the dysbiosis of the lung and gut microbiomes and pregnancy. Respiratory Investigation, 60, 496–502. 35422403 10.1016/j.resinv.2022.03.002 PMC8977498

[phy215761-bib-0004] Barcik, W. , Boutin, R. C. T. , Sokolowska, M. , & Finlay, B. B. (2020). The role of lung and gut microbiota in the pathology of asthma. Immunity, 52, 241–255. 32075727 10.1016/j.immuni.2020.01.007 PMC7128389

[phy215761-bib-0005] Bassis, C. M. , Erb‐Downward, J. R. , Dickson, R. P. , Freeman, C. M. , Schmidt, T. M. , Young, V. B. , Beck, J. M. , Curtis, J. L. , & Huffnagle, G. B. (2015). Analysis of the upper respiratory tract microbiotas as the source of the lung and gastric microbiotas in healthy individuals. MBio, 6, e00037. 25736890 10.1128/mBio.00037-15 PMC4358017

[phy215761-bib-0006] Begley, L. A. , Opron, K. , Bian, G. , Kozik, A. J. , Liu, C. , Felton, J. , Wen, B. , Sun, D. , & Huang, Y. J. (2022). Effects of fluticasone propionate on Klebsiella pneumoniae and gram‐negative bacteria associated with chronic airway disease. mSphere, 7(6), e0037722. 36342141 10.1128/msphere.00377-22 PMC9769713

[phy215761-bib-0007] Blachier, F. , & Andriamihaja, M. (2022). Effects of the L‐tyrosine‐derived bacterial metabolite p‐cresol on colonic and peripheral cells. Amino Acids, 54, 325–338. 34468872 10.1007/s00726-021-03064-x

[phy215761-bib-0008] Campbell, E. M. , Charo, I. F. , Kunkel, S. L. , Strieter, R. M. , Boring, L. , Gosling, J. , & Lukacs, N. W. (1999). Monocyte chemoattractant protein‐1 mediates cockroach allergen‐induced bronchial hyperreactivity in normal but not CCR2−/− mice: The role of mast cells. Journal of Immunology, 163, 2160–2167. 10438957

[phy215761-bib-0009] Chambers, E. S. , Nanzer, A. M. , Pfeffer, P. E. , Richards, D. F. , Martineau, A. R. , Griffiths, C. J. , Corrigan, C. J. , & Hawrylowicz, C. M. (2018). Dendritic cell phenotype in severe asthma reflects clinical responsiveness to glucocorticoids. Clinical and Experimental Allergy, 48, 13–22. 29130617 10.1111/cea.13061 PMC5767735

[phy215761-bib-0010] Chen, R. , Wang, L. , Koch, T. , Curtis, V. , Yin‐DeClue, H. , Handley, S. A. , Shan, L. , Holtzman, M. J. , Castro, M. , & Wang, L. (2020). Sex effects in the association between airway microbiome and asthma. Annals of Allergy, Asthma & Immunology, 125(652–657), 652–657.e3. 10.1016/j.anai.2020.09.007 PMC8043253 32931909

[phy215761-bib-0011] Christiansen, S. C. , & Zuraw, B. L. (2019). Treatment of hypertension in patients with asthma. The New England Journal of Medicine, 381, 1046–1057. 31509675 10.1056/NEJMra1800345

[phy215761-bib-0012] Ciprandi, G. , De Amici, M. , Tosca, M. , & Fuchs, D. (2010). Tryptophan metabolism in allergic rhinitis: The effect of pollen allergen exposure. Human Immunology, 71, 911–915. 20540982 10.1016/j.humimm.2010.05.017

[phy215761-bib-0013] Contoli, M. , Pauletti, A. , Rossi, M. R. , Spanevello, A. , Casolari, P. , Marcellini, A. , Forini, G. , Gnesini, G. , Marku, B. , Barnes, N. , Rizzi, A. , Curradi, G. , Caramori, G. , Morelli, P. , & Papi, A. (2017). Long‐term effects of inhaled corticosteroids on sputum bacterial and viral loads in COPD. The European Respiratory Journal, 50, 1700451. 28982774 10.1183/13993003.00451-2017

[phy215761-bib-0014] Daley‐Yates, P. , Keppler, B. , Baines, A. , Bardsley, G. , & Fingleton, J. (2022). Metabolomic changes related to airway inflammation, asthma pathogenesis and systemic activity following inhaled fluticasone furoate/vilanterol: A randomized controlled trial. Respiratory Research, 23, 258. 36127726 10.1186/s12931-022-02164-w PMC9487108

[phy215761-bib-0015] Dickson, R. P. , Erb‐Downward, J. R. , Falkowski, N. R. , Hunter, E. M. , Ashley, S. L. , & Huffnagle, G. B. (2018). The lung microbiota of healthy mice are highly variable, cluster by environment, and reflect variation in baseline lung innate immunity. American Journal of Respiratory and Critical Care Medicine, 198, 497–508. 29533677 10.1164/rccm.201711-2180OC PMC6118022

[phy215761-bib-0016] Dickson, R. P. , Erb‐Downward, J. R. , Freeman, C. M. , McCloskey, L. , Beck, J. M. , Huffnagle, G. B. , & Curtis, J. L. (2015). Spatial variation in the healthy human lung microbiome and the adapted Island model of lung biogeography. Annals of the American Thoracic Society, 12, 821–830. 25803243 10.1513/AnnalsATS.201501-029OC PMC4590020

[phy215761-bib-0017] Douglas, G. M. , Maffei, V. J. , Zaneveld, J. R. , Yurgel, S. N. , Brown, J. R. , Taylor, C. M. , Huttenhower, C. , & Langille, M. G. I. (2020). PICRUSt2 for prediction of metagenome functions. Nature Biotechnology, 38, 685–688. 10.1038/s41587-020-0548-6 PMC7365738 32483366

[phy215761-bib-0018] Durack, J. , Lynch, S. V. , Nariya, S. , Bhakta, N. R. , Beigelman, A. , Castro, M. , Dyer, A. M. , Israel, E. , Kraft, M. , Martin, R. J. , Mauger, D. T. , Rosenberg, S. R. , Sharp‐King, T. , White, S. R. , Woodruff, P. G. , Avila, P. C. , Denlinger, L. C. , Holguin, F. , Lazarus, S. C. , … Blood Institute's A . (2017). Features of the bronchial bacterial microbiome associated with atopy, asthma, and responsiveness to inhaled corticosteroid treatment. The Journal of Allergy and Clinical Immunology, 140, 63–75. 27838347 10.1016/j.jaci.2016.08.055 PMC5502827

[phy215761-bib-0019] Fazlollahi, M. , Lee, T. D. , Andrade, J. , Oguntuyo, K. , Chun, Y. , Grishina, G. , Grishin, A. , & Bunyavanich, S. (2018). The nasal microbiome in asthma. The Journal of Allergy and Clinical Immunology, 142(834–843), 834–843.e2. 29518419 10.1016/j.jaci.2018.02.020 PMC6123291

[phy215761-bib-0020] Fujiwara, H. (2021). Crosstalk between intestinal microbiota derived metabolites and tissues in allogeneic hematopoietic cell transplantation. Frontiers in Immunology, 12, 703298. 34512627 10.3389/fimmu.2021.703298 PMC8429959

[phy215761-bib-0021] Fujiwara, Y. , Kato, S. , Nesline, M. K. , Conroy, J. M. , DePietro, P. , Pabla, S. , & Kurzrock, R. (2022). Indoleamine 2,3‐dioxygenase (IDO) inhibitors and cancer immunotherapy. Cancer Treatment Reviews, 110, 102461. 36058143 10.1016/j.ctrv.2022.102461 PMC12187009

[phy215761-bib-0022] Gao, P. (2012). Sensitization to cockroach allergen: Immune regulation and genetic determinants. Clinical & Developmental Immunology, 2012, 563760. 22272212 10.1155/2012/563760 PMC3261483

[phy215761-bib-0023] Goleva, E. , Jackson, L. P. , Harris, J. K. , Robertson, C. E. , Sutherland, E. R. , Hall, C. F. , Good, J. T., Jr. , Gelfand, E. W. , Martin, R. J. , & Leung, D. Y. (2013). The effects of airway microbiome on corticosteroid responsiveness in asthma. American Journal of Respiratory and Critical Care Medicine, 188, 1193–1201. 24024497 10.1164/rccm.201304-0775OC PMC3863730

[phy215761-bib-0024] Gostner, J. M. , Becker, K. , Kofler, H. , Strasser, B. , & Fuchs, D. (2016). Tryptophan metabolism in allergic disorders. International Archives of Allergy and Immunology, 169, 203–215. 27161289 10.1159/000445500 PMC5433561

[phy215761-bib-0025] Guida, G. , Bagnasco, D. , Carriero, V. , Bertolini, F. , Ricciardolo, F. L. M. , Nicola, S. , Brussino, L. , Nappi, E. , Paoletti, G. , Canonica, G. W. , & Heffler, E. (2022). Critical evaluation of asthma biomarkers in clinical practice. Frontiers in Medicine, 9, 969243. 36300189 10.3389/fmed.2022.969243 PMC9588982

[phy215761-bib-0026] Han, M. K. , Agusti, A. , Calverley, P. M. , Celli, B. R. , Criner, G. , Curtis, J. L. , Fabbri, L. M. , Goldin, J. G. , Jones, P. W. , Macnee, W. , Make, B. J. , Rabe, K. F. , Rennard, S. I. , Sciurba, F. C. , Silverman, E. K. , Vestbo, J. , Washko, G. R. , Wouters, E. F. , & Martinez, F. J. (2010). Chronic obstructive pulmonary disease phenotypes: The future of COPD. American Journal of Respiratory and Critical Care Medicine, 182, 598–604. 20522794 10.1164/rccm.200912-1843CC PMC6850732

[phy215761-bib-0027] Hartmann, J. E. , Albrich, W. C. , Dmitrijeva, M. , & Kahlert, C. R. (2021). The effects of corticosteroids on the respiratory microbiome: A systematic review. Frontiers in Medicine, 8, 588584. 33777968 10.3389/fmed.2021.588584 PMC7988087

[phy215761-bib-0028] Harusato, A. , Flannigan, K. L. , Geem, D. , & Denning, T. L. (2015). Phenotypic and functional profiling of mouse intestinal antigen presenting cells. Journal of Immunological Methods, 421, 20–26. 25891794 10.1016/j.jim.2015.03.023 PMC4451390

[phy215761-bib-0029] Hizawa, N. (2022). The understanding of asthma pathogenesis in the era of precision medicine. Allergology International, 72, 3–10. 36195530 10.1016/j.alit.2022.09.001

[phy215761-bib-0030] Huang, C. , Ni, Y. , Du, W. , & Shi, G. (2022). Effect of inhaled corticosteroids on microbiome and microbial correlations in asthma over a 9‐month period. Clinical and Translational Science, 15, 1723–1736. 35514165 10.1111/cts.13288 PMC9283747

[phy215761-bib-0031] Huang, Y. J. , & Boushey, H. A. (2015). The microbiome in asthma. The Journal of Allergy and Clinical Immunology, 135, 25–30. 25567040 10.1016/j.jaci.2014.11.011 PMC4287960

[phy215761-bib-0032] Huang, Y. J. , Nariya, S. , Harris, J. M. , Lynch, S. V. , Choy, D. F. , Arron, J. R. , & Boushey, H. (2015). The airway microbiome in patients with severe asthma: Associations with disease features and severity. The Journal of Allergy and Clinical Immunology, 136, 874–884. 26220531 10.1016/j.jaci.2015.05.044 PMC4600429

[phy215761-bib-0033] Jang, S. , Smit, J. , Kallal, L. E. , & Lukacs, N. W. (2013). Respiratory syncytial virus infection modifies and accelerates pulmonary disease via DC activation and migration. Journal of Leukocyte Biology, 94, 5–15. 23293372 10.1189/jlb.0412195 PMC3685016

[phy215761-bib-0034] Klaessens, S. , Stroobant, V. , De Plaen, E. , & Van den Eynde, B. J. (2022). Systemic tryptophan homeostasis. Frontiers in Molecular Biosciences, 9, 897929. 36188218 10.3389/fmolb.2022.897929 PMC9515494

[phy215761-bib-0035] Kozich, J. J. , Westcott, S. L. , Baxter, N. T. , Highlander, S. K. , & Schloss, P. D. (2013). Development of a dual‐index sequencing strategy and curation pipeline for analyzing amplicon sequence data on the MiSeq Illumina sequencing platform. Applied and Environmental Microbiology, 79, 5112–5120. 23793624 10.1128/AEM.01043-13 PMC3753973

[phy215761-bib-0036] Legendre, P. , & Gallagher, E. D. (2001). Ecologically meaningful transformations for ordination of species data. Oecologia, 129, 271–280. 28547606 10.1007/s004420100716

[phy215761-bib-0037] Li, N. , Qiu, R. , Yang, Z. , Li, J. , Chung, K. F. , Zhong, N. , & Zhang, Q. (2017). Sputum microbiota in severe asthma patients: Relationship to eosinophilic inflammation. Respiratory Medicine, 131, 192–198. 28947029 10.1016/j.rmed.2017.08.016

[phy215761-bib-0038] Licari, A. , Fuchs, D. , Marseglia, G. , & Ciprandi, G. (2019). Tryptophan metabolic pathway and neopterin in asthmatic children in clinical practice. Italian Journal of Pediatrics, 45, 114. 31455379 10.1186/s13052-019-0699-6 PMC6712831

[phy215761-bib-0039] Lukacs, N. W. , Tekkanat, K. K. , Berlin, A. , Hogaboam, C. M. , Miller, A. , Evanoff, H. , Lincoln, P. , & Maassab, H. (2001). Respiratory syncytial virus predisposes mice to augmented allergic airway responses via IL‐13‐mediated mechanisms. Journal of Immunology, 167, 1060–1065. 10.4049/jimmunol.167.2.1060 11441116

[phy215761-bib-0040] Lyu, Z. , Schmidt, R. R. , Martin, R. E. , Green, M. T. , Kinkade, J. A. , Mao, J. , Bivens, N. J. , Joshi, T. , & Rosenfeld, C. S. (2022). Long‐term effects of developmental exposure to oxycodone on gut microbiota and relationship to adult behaviors and metabolism. mSystems, 7, e0033622. 35862801 10.1128/msystems.00336-22 PMC9426609

[phy215761-bib-0041] Ma, C. , Azad, M. A. K. , Tang, W. , Zhu, Q. , Wang, W. , Gao, Q. , & Kong, X. (2022). Maternal probiotics supplementation improves immune and antioxidant function in suckling piglets via modifying gut microbiota. Journal of Applied Microbiology, 133, 515–528. 35396768 10.1111/jam.15572

[phy215761-bib-0042] Malinczak, C. A. , Fonseca, W. , Rasky, A. J. , Ptaschinski, C. , Morris, S. , Ziegler, S. F. , & Lukacs, N. W. (2019). Sex‐associated TSLP‐induced immune alterations following early‐life RSV infection leads to enhanced allergic disease. Mucosal Immunology, 12, 969–979. 31076663 10.1038/s41385-019-0171-3 PMC6599479

[phy215761-bib-0043] Martin, M. J. , Zain, N. M. M. , Hearson, G. , Rivett, D. W. , Koller, G. , Wooldridge, D. J. , Rose, G. , Gharbia, S. E. , Forbes, B. , Bruce, K. D. , & Harrison, T. W. (2020). The airways microbiome of individuals with asthma treated with high and low doses of inhaled corticosteroids. PLoS One, 15, e0244681. 33378384 10.1371/journal.pone.0244681 PMC7773270

[phy215761-bib-0044] Mason, K. L. , Erb Downward, J. R. , Mason, K. D. , Falkowski, N. R. , Eaton, K. A. , Kao, J. Y. , Young, V. B. , & Huffnagle, G. B. (2012). Candida albicans and bacterial microbiota interactions in the cecum during recolonization following broad‐spectrum antibiotic therapy. Infection and Immunity, 80, 3371–3380. 22778094 10.1128/IAI.00449-12 PMC3457555

[phy215761-bib-0045] Miller, A. L. , Bowlin, T. L. , & Lukacs, N. W. (2004). Respiratory syncytial virus‐induced chemokine production: Linking viral replication to chemokine production in vitro and in vivo. The Journal of Infectious Diseases, 189, 1419–1430. 15073679 10.1086/382958

[phy215761-bib-0046] Narayanan, S. , Elesela, S. , Rasky, A. J. , Morris, S. H. , Kumar, S. , Lombard, D. , & Lukacs, N. W. (2022). ER stress protein PERK promotes inappropriate innate immune responses and pathogenesis during RSV infection. Journal of Leukocyte Biology, 111, 379–389. 33866604 10.1002/JLB.3A0520-322RR

[phy215761-bib-0047] Nguyen, T. T. T. , Oshima, K. , Toh, H. , Khasnobish, A. , Fujii, Y. , Arakawa, K. , & Morita, H. (2019). Draft genome sequence of Butyricimonas faecihominis 30A1, isolated from feces of a Japanese Alzheimer's disease patient. Microbiology Resource Announcements, 8, 8. 10.1128/MRA.00462-19 PMC6554612 31171624

[phy215761-bib-0048] Noverr, M. C. , Noggle, R. M. , Toews, G. B. , & Huffnagle, G. B. (2004). Role of antibiotics and fungal microbiota in driving pulmonary allergic responses. Infection and Immunity, 72, 4996–5003. 15321991 10.1128/IAI.72.9.4996-5003.2004 PMC517468

[phy215761-bib-0049] Pang, Z. , Wang, G. , Gibson, P. , Guan, X. , Zhang, W. , Zheng, R. , Chen, F. , Wang, Z. , & Wang, F. (2019). Airway microbiome in different inflammatory phenotypes of asthma: A cross‐sectional study in Northeast China. International Journal of Medical Sciences, 16, 477–485. 30911282 10.7150/ijms.29433 PMC6428974

[phy215761-bib-0050] Passarelli, A. , Pisano, C. , Cecere, S. C. , Di Napoli, M. , Rossetti, S. , Tambaro, R. , Ventriglia, J. , Gherardi, F. , Iannacone, E. , Venanzio, S. S. , Fiore, F. , Bartoletti, M. , Scognamiglio, G. , Califano, D. , & Pignata, S. (2022). Targeting immunometabolism mediated by the IDO1 pathway: A new mechanism of immune resistance in endometrial cancer. Frontiers in Immunology, 13, 953115. 36119020 10.3389/fimmu.2022.953115 PMC9479093

[phy215761-bib-0051] Patel, P. S. , King, R. G. , & Kearney, J. F. (2016). Pulmonary alpha‐1,3‐glucan‐specific IgA‐secreting B cells suppress the development of cockroach allergy. Journal of Immunology, 197, 3175–3187. 10.4049/jimmunol.1601039 PMC5101147 27581173

[phy215761-bib-0052] Pruteanu, A. I. , Chauhan, B. F. , Zhang, L. , Prietsch, S. O. , & Ducharme, F. M. (2014). Inhaled corticosteroids in children with persistent asthma: Dose‐response effects on growth. Cochrane Database of Systematic Reviews, 2014, CD009878. 25030199 10.1002/14651858.CD009878.pub2 PMC8932085

[phy215761-bib-0053] Raftis, E. J. , Delday, M. I. , Cowie, P. , McCluskey, S. M. , Singh, M. D. , Ettorre, A. , & Mulder, I. E. (2018). Bifidobacterium breve MRx0004 protects against airway inflammation in a severe asthma model by suppressing both neutrophil and eosinophil lung infiltration. Scientific Reports, 8, 12024. 30104645 10.1038/s41598-018-30448-z PMC6089914

[phy215761-bib-0054] Schloss, P. D. , Westcott, S. L. , Ryabin, T. , Hall, J. R. , Hartmann, M. , Hollister, E. B. , Lesniewski, R. A. , Oakley, B. B. , Parks, D. H. , Robinson, C. J. , Sahl, J. W. , Stres, B. , Thallinger, G. G. , Van Horn, D. J. , & Weber, C. F. (2009). Introducing mothur: Open‐source, platform‐independent, community‐supported software for describing and comparing microbial communities. Applied and Environmental Microbiology, 75, 7537–7541. 19801464 10.1128/AEM.01541-09 PMC2786419

[phy215761-bib-0055] Seekatz, A. M. , Theriot, C. M. , Molloy, C. T. , Wozniak, K. L. , Bergin, I. L. , & Young, V. B. (2015). Fecal microbiota transplantation eliminates Clostridium difficile in a murine model of relapsing disease. Infection and Immunity, 83, 3838–3846. 26169276 10.1128/IAI.00459-15 PMC4567621

[phy215761-bib-0056] Segata, N. , Izard, J. , Waldron, L. , Gevers, D. , Miropolsky, L. , Garrett, W. S. , & Huttenhower, C. (2011). Metagenomic biomarker discovery and explanation. Genome Biology, 12, R60. 21702898 10.1186/gb-2011-12-6-r60 PMC3218848

[phy215761-bib-0057] Sohn, M. H. , & Kim, K. E. (2012). The cockroach and allergic diseases. Allergy, Asthma & Immunology Research, 4, 264–269. 10.4168/aair.2012.4.5.264 PMC3423599 22950031

[phy215761-bib-0058] Tchitchek, N. , Nguekap Tchoumba, O. , Pires, G. , Dandou, S. , Campagne, J. , Churlaud, G. , Fourcade, G. , Hoffmann, T. W. , Strozzi, F. , Gaal, C. , Bonny, C. , Le Chatelier, E. , Erlich, S. D. , Sokol, H. , & Klatzmann, D. (2022). Low‐dose IL‐2 shapes a tolerogenic gut microbiota that improves autoimmunity and gut inflammation. JCI Insight, 7, e159406. 35917175 10.1172/jci.insight.159406 PMC9536277

[phy215761-bib-0059] Vacca, M. , Celano, G. , Calabrese, F. M. , Portincasa, P. , Gobbetti, M. , & De Angelis, M. (2020). The controversial role of human gut Lachnospiraceae. Microorganisms, 8, 8. 10.3390/microorganisms8040573 PMC7232163 32326636

[phy215761-bib-0060] Wang, J. , Chai, J. , Zhang, L. , Zhang, L. , Yan, W. , Sun, L. , Chen, Y. , Sun, Y. , Zhao, J. , & Chang, C. (2021). Microbiota associations with inflammatory pathways in asthma. Clinical and Experimental Allergy, 52, 697–705. 10.1111/cea.14089 34962671

[phy215761-bib-0061] Wang, Q. , Li, F. , Liang, B. , Liang, Y. , Chen, S. , Mo, X. , Ju, Y. , Zhao, H. , Jia, H. , Spector, T. D. , Xie, H. , & Guo, R. (2018). A metagenome‐wide association study of gut microbiota in asthma in UK adults. BMC Microbiology, 18, 114. 30208875 10.1186/s12866-018-1257-x PMC6134768

[phy215761-bib-0062] Wang, Z. , Lai, Z. , Zhang, X. , Huang, P. , Xie, J. , Jiang, Q. , Zhang, Q. , & Chung, K. F. (2021). Altered gut microbiome compositions are associated with the severity of asthma. Journal of Thoracic Disease, 13, 4322–4338. 34422359 10.21037/jtd-20-2189 PMC8339736

[phy215761-bib-0063] Yagi, K. , Huffnagle, G. B. , Lukacs, N. W. , & Asai, N. (2021). The lung microbiome during health and disease. International Journal of Molecular Sciences, 22(19), 10872. 34639212 10.3390/ijms221910872 PMC8509400

[phy215761-bib-0064] Yip, W. , Li, X. , Koelwyn, G. J. , Milne, S. , Leitao Filho, F. S. , Yang, C. X. , Hernandez Cordero, A. I. , Yang, J. , Yang, C. W. T. , Shaipanich, T. , van Eeden, S. F. , Leung, J. M. , Lam, S. , McNagny, K. M. , & Sin, D. D. (2022). Inhaled corticosteroids selectively Alter the microbiome and host transcriptome in the small Airways of Patients with chronic obstructive pulmonary disease. Biomedicine, 10, 10. 10.3390/biomedicines10051110 PMC9138653 35625847

[phy215761-bib-0065] Zhang, J. , Ma, J. , Li, Q. , Su, H. , & Sun, X. (2021). Exploration of the effect of mixed probiotics on microbiota of allergic asthma mice. Cellular Immunology, 367, 104399. 34192623 10.1016/j.cellimm.2021.104399

[phy215761-bib-0066] Zhang, Q. , Cox, M. , Liang, Z. , Brinkmann, F. , Cardenas, P. A. , Duff, R. , Bhavsar, P. , Cookson, W. , Moffatt, M. , & Chung, K. F. (2016). Airway microbiota in severe asthma and relationship to asthma severity and phenotypes. PLoS One, 11, e0152724. 27078029 10.1371/journal.pone.0152724 PMC4831690

[phy215761-bib-0067] Zhao, Q. , Wu, Z. E. , Li, B. , & Li, F. (2022). Recent advances in metabolism and toxicity of tyrosine kinase inhibitors. Pharmacology & Therapeutics, 237, 108256. 35901905 10.1016/j.pharmthera.2022.108256

[phy215761-bib-0068] Zheng, J. , Wu, Q. , Zhang, L. , Zou, Y. , Wang, M. , He, L. , & Guo, S. (2022). Anti‐inflammatory activities of Qingfei oral liquid and its influence on respiratory microbiota in mice with ovalbumin‐induced asthma. Frontiers in Pharmacology, 13, 911667. 36081945 10.3389/fphar.2022.911667 PMC9445488

[phy215761-bib-0069] Zheng, J. , Wu, Q. , Zou, Y. , Wang, M. , He, L. , & Guo, S. (2021). Respiratory microbiota profiles associated with the progression from airway inflammation to remodeling in mice with OVA‐induced asthma. Frontiers in Microbiology, 12, 723152. 34526979 10.3389/fmicb.2021.723152 PMC8435892

[phy215761-bib-0070] Zheng, T. J. , Parra‐Izquierdo, I. , Reitsma, S. E. , Heinrich, M. C. , Larson, M. K. , Shatzel, J. J. , Aslan, J. E. , & McCarty, O. J. T. (2022). Platelets and tyrosine kinase inhibitors: Clinical features, mechanisms of action, and effects on physiology. American Journal of Physiology. Cell Physiology, 323, C1231–C1250. 35938677 10.1152/ajpcell.00040.2022 PMC9576167

[phy215761-bib-0071] Zou, X. L. , Wu, J. J. , Ye, H. X. , Feng, D. Y. , Meng, P. , Yang, H. L. , Wu, W. B. , Li, H. T. , He, Z. , & Zhang, T. T. (2021). Associations between gut microbiota and asthma Endotypes: A cross‐sectional study in South China based on patients with newly diagnosed asthma. Journal of Asthma and Allergy, 14, 981–992. 34408443 10.2147/JAA.S320088 PMC8367087

