# Supplementary material for: A steroid‐resistant cockroach allergen model is associated with lung and cecal microbiome changes
Source: Physiol Rep. 2023 Jul 4;11(13):e15761. doi: 10.14814/phy2.15761 (PMC10320043; doi:10.14814/phy2.15761)
Supplement: Supplementary file 1 — Figure S1: [file PHY2-11-e15761-s001.docx]

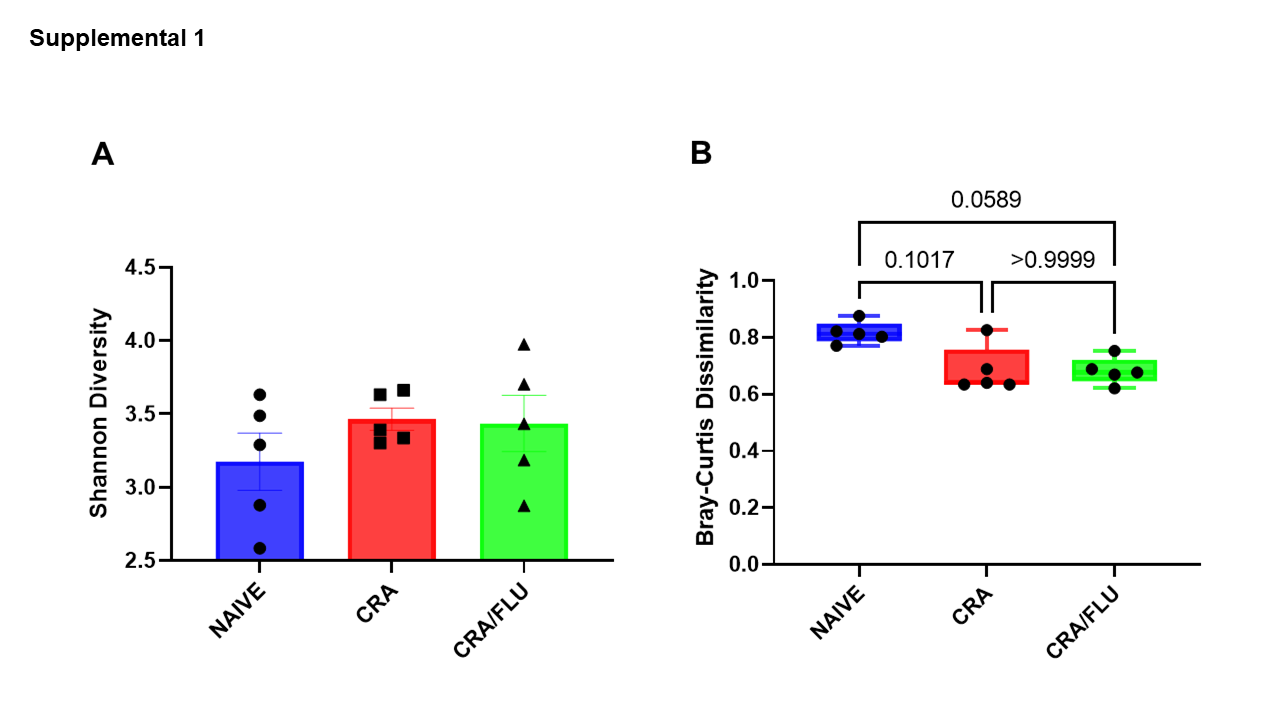


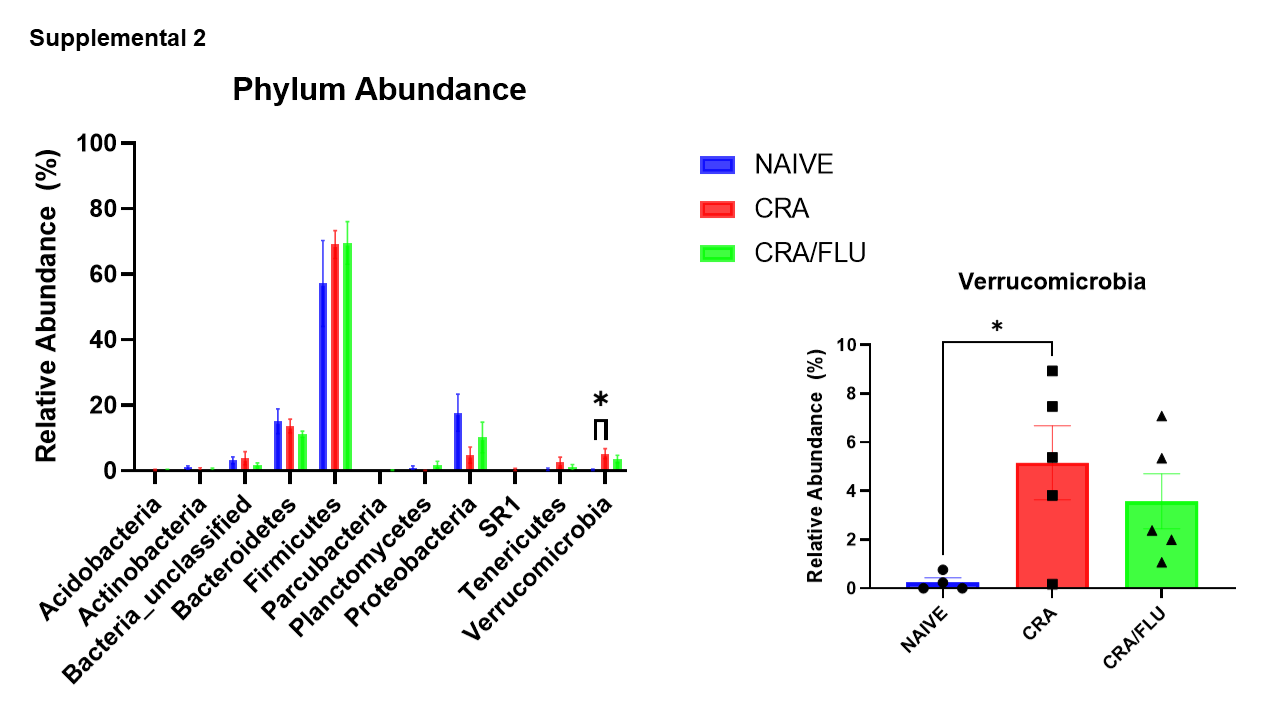


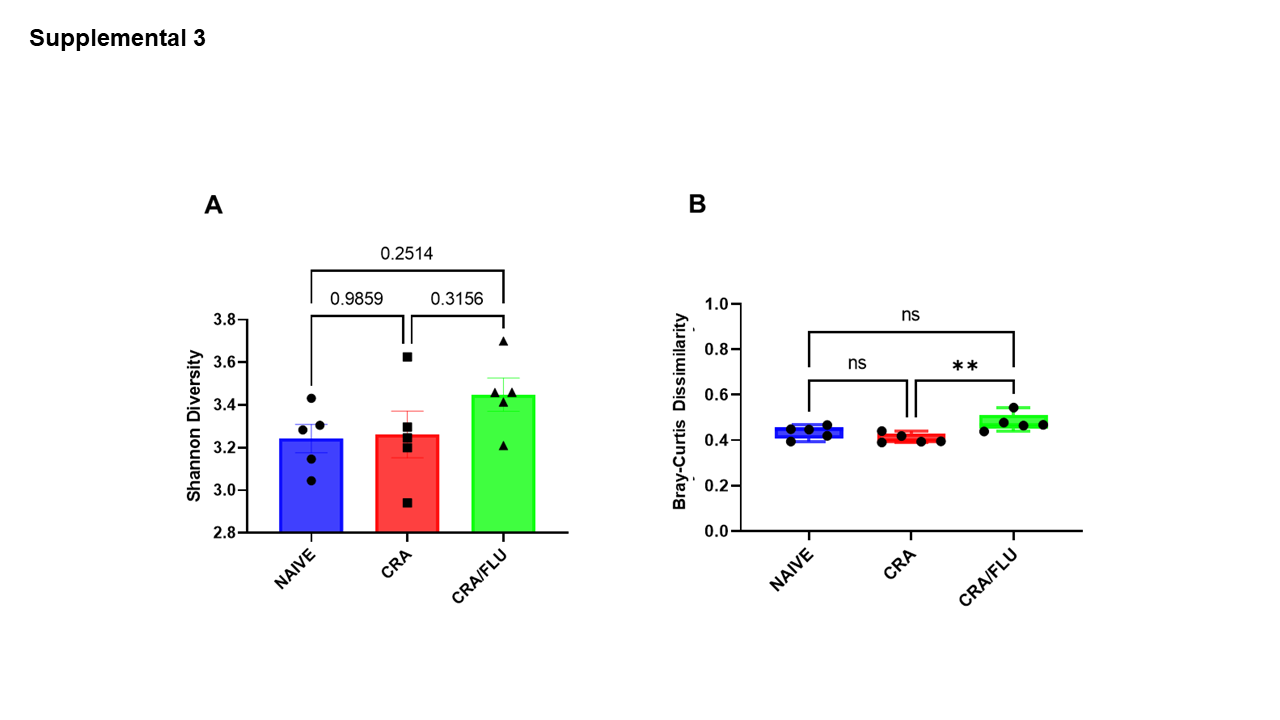


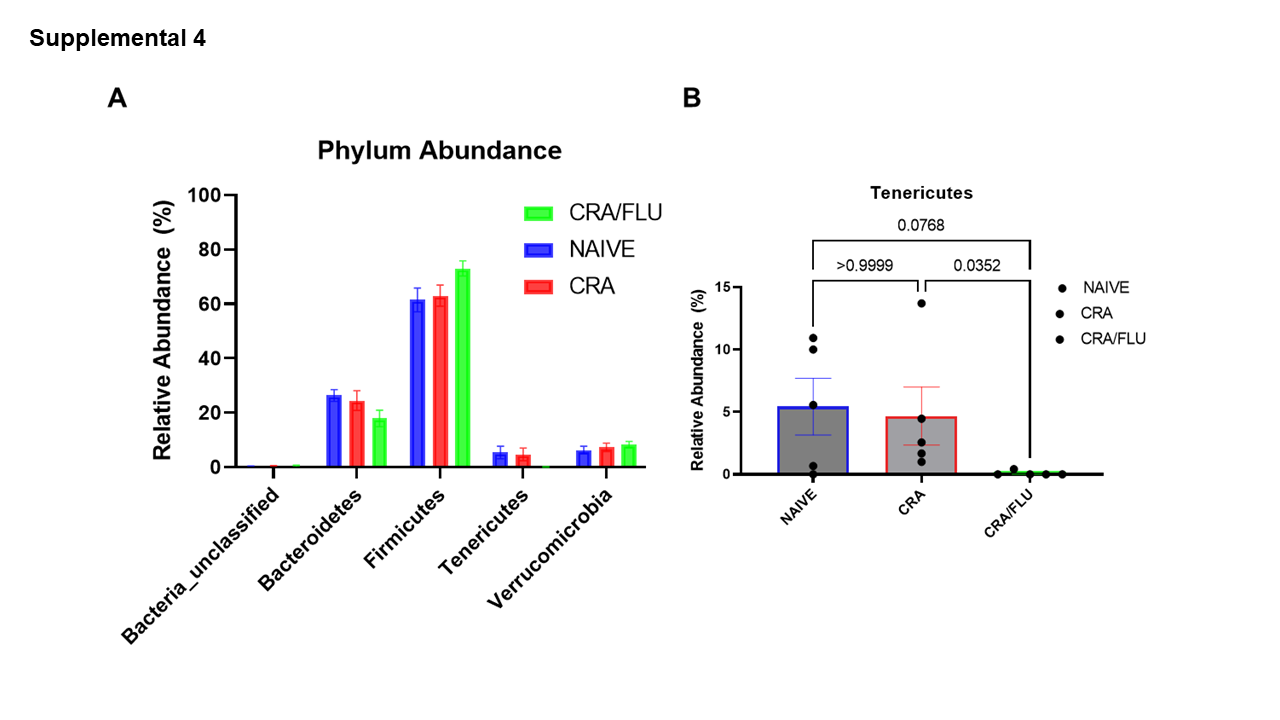


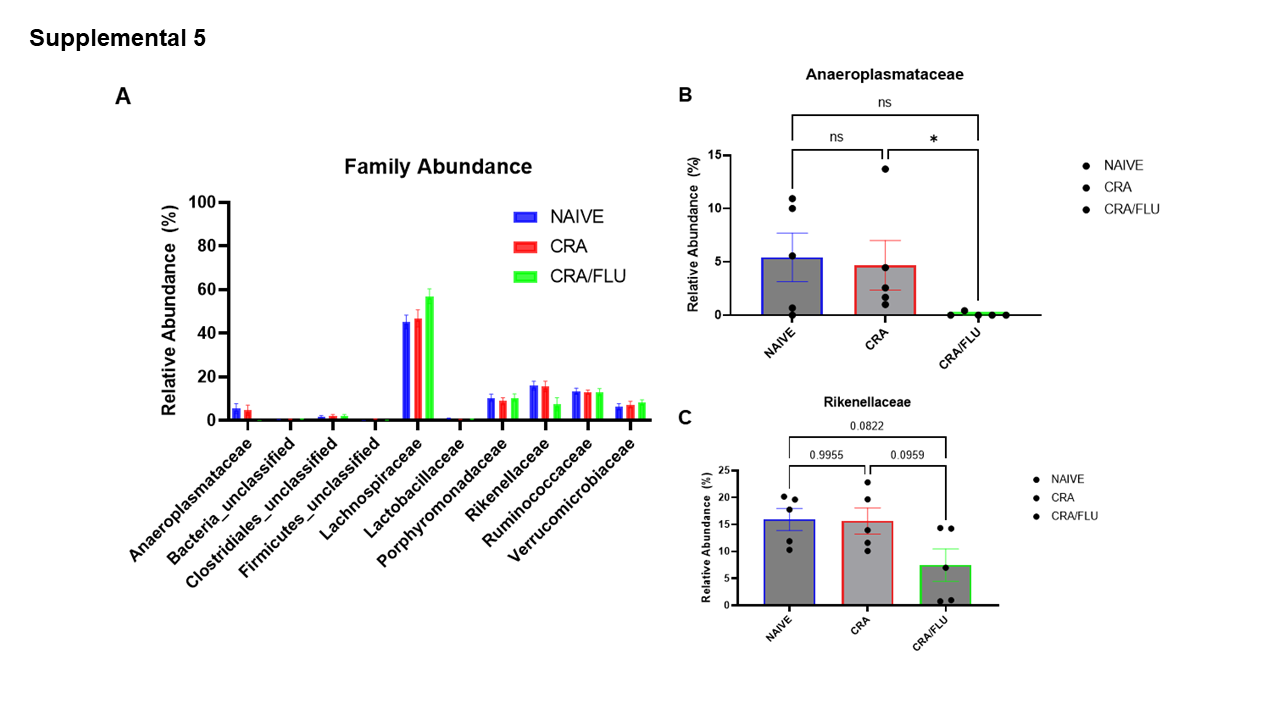


**Supplemental Figure Legends-**

Supplemental Figure 1- The analysis of microbial composition changes in the lung was measured using A) Shannon diversity index and B) Bray-Curtis dissimilarity index.

Supplemental Figure 2- Examination of the microbial composition of the lung microbiome at the phylum level between the Naïve, CRA and CRA with fluticasone treatment (CRA/FLU). The only significant difference was an increase with Verricumicrobia in the allergen sensitized and challenged mice.

Supplemental Figure 3- The analysis of microbial composition changes in the cecum was measured using A) Shannon diversity index and B) Bray-Curtis dissimilarity index.

Supplemental Figure 4- Examination of the microbial composition of the lung microbiome at the phylum level between the Naïve, CRA and CRA with fluticasone treatment (CRA/FLU). The only significant difference was a decrease in Ternericutes in the allergic mice treated with fluticasone (CRA/FLU).

Supplemental Figure 5- As the cecum had higher biomass our analyses were able to examine differences in composition at the family level. The data demonstrate decreased abundance of Anaeroplasmataceae and Rikenellaceae in the CRA/FLU group.
